# The *in vitro* and *in vivo* Antioxidant and Immunomodulatory Activity of Incomplete Degradation Products of Hemicellulosic Polysaccharide (Galactomannan) From *Sesbania cannabina*

**DOI:** 10.3389/fbioe.2021.679558

**Published:** 2021-04-09

**Authors:** Yuheng Tao, Ting Wang, Chenhuan Lai, Zhe Ling, Yanmin Zhou, Qiang Yong

**Affiliations:** ^1^Jiangsu Co-Innovation Center for Efficient Processing and Utilization of Forest Resources, Nanjing Forestry University, Nanjing, China; ^2^Key Laboratory of Forest Genetics and Biotechnology of Ministry of Education, Nanjing Forestry University, Nanjing, China; ^3^College of Animal Science and Technology, Nanjing Agricultural University, Nanjing, China

**Keywords:** hemicellulosic polysaccharide, incomplete degradation products of galactomannan, antioxidant function, immunomodulatory activity, *Sesbania cannabina*

## Abstract

As known, the nutritional status affects antioxidant capacity and immunity, ultimately affecting the body’s health. Recently, hemicellulosic polysaccharides of galactomannan in different biomass and their degradation products are gaining more attention due to excellent antioxidant enhancement and immunomodulatory activity. Herein, incomplete degradation products of galactomannan (IDPG) were prepared from the enzymatic hydrolysis of *Sesbania cannabina* seeds, followed by the *in vitro* and *in vivo* experiments. Using an H_2_O_2_-injured RAW264.7 cells model, IDPG was demonstrated to have antioxidant capacity, as indicated by superoxide dismutase (SOD) activity and malondialdehyde (MDA) content. While in the evaluation in laying hens (68-weeks-old), diets were supplemented with 0, 0.01, 0.025, and 0.05% IDPG for 8 weeks, respectively. Our results showed that IDPG can improve antioxidant capacity by increasing antioxidants contents and reducing MDA content. Furthermore, IDPG can increase immunoglobulins and cytokines secretion, thereby enhancing the immunity of laying hens. This result was further demonstrated by *in vitro* experiment, in which IDPG significantly increased the secretion of nitric oxide (NO), tumor necrosis factor-α (TNF-α), interleukin 6 (IL-6), and toll-like receptor 4 (TLR4) in RAW264.7 cells (*P* < 0.05). Overall, IDPG can improve antioxidant function and modulate immunological response, thereby the concept of using IDPG for health may gain a little more credibility.

## Introduction

Hemicellulosic polysaccharides from different biomass have been applied in various fields (Li et al., 2020; Xiang et al., 2020). Recently, numerous *in vitro* experiments about natural polysaccharides from different biomass have confirmed that they can not only enhance immunity but also suppress excessive immune responses caused by various stimuli (Tang et al., 2019). This subset of polysaccharides includes examples such as arabinogalactans (Tang et al., 2018), galactomannan (Gu et al., 2020), β-glucan (Pan et al., 2020), and so on. Among these examples, polysaccharides with mannose as the main chain, such as yeast cell wall mannan, glucomannan, and galactomannan, have garnered great interest because they are easier to bind several receptors on immune cells to activate immune responses (Hernandez et al., 2011). Toll-like receptor 4 (TLR4), an important receptor in both innate and adaptive immune responses, was identified to have a high affinity for acemannan (Karaca et al., 1995). Moreover, mannose-binding lectins present on macrophages can bind mannan and activate the immunity via a non-self-recognition mechanism (Gamal-Eldeen et al., 2006). These outstanding characteristics constitute the major advantages of polysaccharides with mannose as the main chain different from other types of polysaccharides on immunity function. Besides, mannan or galactomannan has been found to have beneficial antioxidant functionality. For example, galactomannan from *Caesalpinia gilliesii* was found to induce a significant reduction in hepatic malondialdehyde in Wister albino rats (Abdel-Megeed et al., 2019). Based on these properties, these multifunctional materials can be further applied *in vivo* experimentation as an animal feed additive.

Considering the entire array of components involved in the immune system, it presents as a complex, but precisely interwoven network of biochemical mechanisms (Devasagayam and Sainis, 2002). It is vulnerable to oxidative stress from reactive oxygen, which attacks cellular components produced during the functioning of the immune system and leads immune cells to death (De la Fuente, 2002; Pei et al., 2020; Yu et al., 2020; Gu et al., 2021; Zheng et al., 2021). Therefore, during certain diseased states or aging, there is a need for enhancing the antioxidant capacity while potentiating the immune function. In this concept immunomodulators having antioxidant abilities, especially natural polysaccharides have considerable potential.

The attention was put on the galactomannan from the endosperm of *Sesbania cannabina* seeds, which is widely available in many coastal regions of tropical and subtropical countries of Asia (Hossain et al., 2002). The tolerance of *S. cannabina* to salt and barren accompanies by growing quickly allows it can grow in poor soil (Cowan et al., 1982). But for too long, the *S. cannabina* seeds have lacked an effective use. A promising use as a plant-based protein source is also no longer valued due to the reduction in nutrient absorption caused by the high viscosity of galactomannan. Hossain et al. (2001) found that the inclusion of *S. aculeate* seeds in the diet of common carp (*Cyprinus carpio*) affects nutrient absorption and utilization. Fortunately, this side effect can be erased by lowering the molecular weight of galactomannan, because an example of partially hydrolyzed guar gum (PHGG) was reported. The supplement of 5% intact guar gum (mainly galactomannan) in the diet of rats decreased food consumption, resulting in a lower increase in body weight gain, as described by Hidehisa et al. (1994). However, the rats fed on 5% PHGG did not show any significant change in the above values relative to the control diet. Based on this successful practice, it is possible to concentrate on the effect of incomplete degradation products of galactomannan (IDPG) on the antioxidant capacity and immune function of laying hens after eliminating the possible negative effects brought by viscosity.

Generally speaking, the IDPG refer to the degradation products of natural galactomannan with a polymerization degree of more than 2, which do not contain monosaccharides such as mannose and galactose. Herein, an enzymatic degradation process was performed by β-endo-mannanase to obtain IDPG (Tao et al., 2020). Specifically, the hydrolysis effect of mannanase reduced the average molecular weight of galactomannan to one-tenth of the initial value. Next, the anti-oxidative ability of IDPG was evaluated by analyzing the cellular protective effects against H_2_O_2_-induced injury in macrophage RAW 264.7 cells. Moreover, the immunomodulatory properties of IDPG were evaluated by analyzing their ability on influencing the secretion of NO and cytokines from macrophage RAW 264.7 cells. Furthermore, a feeding trial involving laying hens was conducted to evaluate how varied IDPG feed addition rates influence antioxidant capacity and immune function. It is our intent for this work to provide new evidence supporting the value in both the preparation and utilization of IDPG for laying hen farming. Moreover, the development of high value-added IDPG to feed additives will increase the economic value of *S. cannabina* seeds.

## Materials and Methods

### Preparation of the IDPG

The IDPG was prepared by enzymatic hydrolysis of galactomannan from *Sesbania cannabina* seeds using β-mannanase. The *Sesbania cannabina* seeds used in this experiment were purchased from a local farm in Yancheng city, Jiangsu Province of China. First, *S. cannabina* seeds were ground (Mini plant shredder F2102, Taisite Instrument Co., Ltd., Tianjin, China) and then suspended in distilled water at a galactomannan concentration of 40 g/L. The suspension was treated with β-mannanase from *Trichoderma reesei* (20 U/g galactomannan, 72 h, 50°C). β-endo-mannanase (EC 3.2.1.78) was obtained from *T. reesei* Rut C-30 using avicel as a substrate. Before adding an enzyme, the pH of the enzyme treatment solution was adjusted to 4.8 with 0.05 M citric acid buffer. After reaction time ceased, enzyme deactivation was applied by boiling the mixture at ∼100°C for 10 min. Once boiled, the suspension was centrifuged (10,000 rpm, 10 min) and the obtained supernatants were nanofiltrated (200 Da, ST-Recovery Tech Co., Nanjing, Jiangsu, China) to remove galactose and mannose and henceforth referred to as IDPG solutions. Finally, this solution was spray-dried at 160°C (B-191, BUCHI, Flawil, Switzerland) to obtain solid IDPG. The galactomannan degradation products content in the solid IDPG was 45.96%, which was determined by a sulfuric acid hydrolysis method and high-performance anion-exchange chromatography with pulsed amperometric detection as described by Tao et al. (2020). The weight-average molecular weight of IDPG was finally determined to 1.74∼14.12 kDa by high-performance size exclusion chromatography (HPSEC) as described by Tao et al. (2020).

### Antioxidant Activity Evaluated by H_2_O_2_-Induced Injury Cell Model

#### Cell Culture

The cells were cultured in DMEM supplemented with penicillin (100 units/mL), streptomycin (100 units/mL), and 10% (v/v) fetal bovine serum (FBS) at 37°C in a humidified atmosphere with 5% CO_2_.

#### Toxicity Measurements

The toxicity measurements of IDPG on the RAW 264.7 cells were evaluated by cultivating cells with IDPG at different concentrations (25, 50, 100, 200, 400, and 800 μg/mL) for 24 h. Toxicity results were expressed as the cell viability, which was determined by a CCK-8 assay according to the manufacturer’s protocols. All toxicity experiments were performed in triplicates.

#### Injured Cell Model Induced by H_2_O_2_

RAW 264.7 cells were seeded on a culture of 96-well plates at a density of 1 × 10^5^ cells/mL and cultivated for 24 h. After removing the medium, a new medium containing H_2_O_2_ (50∼3200 μM) was added and incubated for 4 h. The group treated with fresh complete medium without H_2_O_2_ for 4 h was taken as a control. Assay results were expressed as the cell viability determined by a CCK-8 assay according to the manufacturer’s protocols. All experiments were performed in triplicate.

#### Protective Effects of IDPG Against H_2_O_2_-Induced Cellular Injury

RAW 264.7 cells were first exposed to IDPG at different concentrations (25, 50, 100, 200, 400, and 800 μg/mL) for 24 h. Then, each treatment was exposed to a medium containing H_2_O_2_ for another 4 h, while the control group was replaced with a fresh complete medium for 4 h (Wang et al., 2015). Moreover, the H_2_O_2_ model group was incubated complete medium for 24 h and then treated with a new medium containing H_2_O_2_ for another 4 h. According to the manufacturer’s protocols, results were expressed as the cell viability, as determined by a CCK-8 assay. All these experiments were performed in triplicate.

#### Determination of SOD Activity and MDA Content

RAW264.7 cells were cultivated with IDPG at different concentrations (25, 50, 100, 200, 400, and 800 μg/mL) for 24 h. Next, the H_2_O_2_ model group and IDPG groups were exposed to H_2_O_2_ (2.4 mM) for 4 h, and cells were next dissociated for measurements of the activity of superoxide dismutase (SOD) and lipid peroxidation (MDA). Both the SOD activity and MDA content were evaluated by using kits according to the manufacturer’s protocols. SOD activity was analyzed by the hydroxylamine method (Oyanagui, 1984). One unit of SOD is defined as the amount of enzyme per milligram of protein required to produce 50% inhibition of the rate of nitrite production at 37°C. The MDA content was determined by barbiturate thiosulfate assay (Placer et al., 1966) and expressed in nmol/mg protein. The protein concentration was also measured by a BCA protein assay kit according to the manufacturer’s protocols. The absorbance was determined by the microplate reader (FilterMax F5, Molecular Devices, Sunnyvale, CA, United States). All these experiments were also performed in triplicate.

### Immunomodulatory Activity Evaluated by RAW264.7 Cells

RAW 264.7 cells (1 × 10^5^ cells/well) were loaded into a 6-well plate and then cultured with different concentrations of IDPG or LPS (1 μg/ml) for another 24 h. After centrifugation of medium, the obtained supernatants were collected and the production of nitric oxide (NO), tumor necrosis factor-α (TNF-α), interleukin 6 (IL-6), and interleukin 1β (IL-1β) were measured by kits according to the manufacturer’s protocols. All these experiments were performed in triplicate.

### Effect of IDPG on the Antioxidant Capacity and Immune Function of Laying Hens

#### Animal Care and Experimental Design

The experimental protocols used in this experiment, including animal care and use, were reviewed and approved by the Animal Care and Use Ethics Committee of Nanjing Agricultural University (Nanjing, China). A total of 288 68-weeks-old laying hens (Hy-Line variety brown) were randomly distributed into four dietary treatments consisting of six replicates (cages) with 12 birds per replicate. The four groups were fed a basal diet supplemented with 0 (control group), 0.01, 0.025, and 0.05% IDPG for 8 weeks. The ingredients composition and nutrients content of the basal diet are given in Table 1. Kept inside of an environmentally controlled house, the birds were allowed free access to water and mash feed in 3-level cages (120 cm × 60 cm × 50 cm; 0.09 m^2^ per chick) with controlled ventilation and lighting (16L:8D). The laying hens were fed twice per day (6 AM and 3 PM), and the feed was mixed into the trough to ensure its full consumption. According to the feeding situation of the day before, the feeding amount for the proceeding day was appropriately adjusted to ensure that there was no remaining feed in the trough each night. After 2 weeks of preliminary testing, a formal experiment was then initiated and carried out for the next 8 weeks.

**TABLE 1:** Composition and nutrient levels of basal diets (as-fed basis).

Ingredients	Content (%)	Nutrient levels^b^	
Corn	63.50	Apparent metabolizable energy (MJ/kg)	11.16
Soybean meal	18.80	Crude protein (%)	15.37
Fish meal	1.50	Calcium (%)	3.79
Rapeseed meal	2.00	Total phosphorus (%)	0.64
Corn gluten meal	1.20		
Soybean phospholipid	1.00		
Limestone	9.17		
Dicalcium phosphate	1.40		
DL-Methionine	0.10		
Sodium chloride	0.33		
1% Premix^a^	1.00		
Total	100		

*^*a*^1% Premix was provided by Huamu Institute of Animal Science and Technology, and provided per kilogram of diet: vitamin A (transretinyl acetate), 1.08 × 10^4^ IU; vitamin D3 (cholecalciferol), 2.7 × 10^3^ IU; vitamin E (all-rac-α-tocopherol), 27 mg; menadione, 0.84 mg; thiamin, 0.72 mg; riboflavin, 5.4 mg; nicotinamide, 9 mg; calcium pantothenate, 36 mg; pyridoxine^⋅^HCl, 2.7 mg; biotin, 0.09 mg; folic acid, 0.24 mg; vitamin B12 (cobalamin), 0.009 mg; Fe (from ferrous sulfate), 100 mg; Cu (from copper sulfate), 8.0 mg; Mn (from manganese sulfate), 100 mg; Zn (from zinc oxide), 60 mg; I (from calcium iodate), 0.9 mg; and Se (from sodium selenite), 0.3 mg. ^*b*^Nutrient levels were the calculated values.*

#### Sample Collection

At 76 weeks of age, a total of 24 birds from all treatments were randomly selected (one bird per replicate). Blood samples were collected from wing veins using sterilized needles and syringes. Samples were then centrifuged at 3,000 rpm for 15 min at 4°C to separate the serum. Obtained serum was collected in new tubes and stored at −20°C until further analysis. The randomly selected laying hens were also euthanized by cervical dislocation and necropsied immediately. Their whole gastrointestinal tract was rapidly removed and placed on a chilled stainless-steel tray. The liver was quickly excised and then rapidly frozen in liquid nitrogen. The organ was then stored at −80°C for further analysis. Both the jejunum (from the end of the pancreatic loop to the Meckel’s diverticulum) and ileum (from Meckel’s diverticulum to the junction of ileocecal) were also separated without the mesentery. Next, the jejunum and ileum were opened longitudinally and the digestive tract was flushed with ice-cold phosphate buffer solution. Subsequently, the jejunal and ileal mucosa were carefully scratched using a sterile glass microscope slide, which was then rapidly frozen in liquid nitrogen and stored at −80°C for further analysis.

#### Determination of the Serum Parameters

The SOD, glutathione peroxidase (GSH-Px), total antioxidant capacity (T-AOC), and MDA were determined using commercial diagnostic kits (Nanjing Jiancheng Bioengineering Institute, Nanjing, Jiangsu, China) according to the manufacturer’s instructions. The SOD activity and MDA concentration were expressed in U/mL serum and mmol/mL of serum, respectively. The ferric-reducing power assay was conducted to determine total antioxidant capacity in serum, and results are expressed in nmol/L of serum (Benzie and Strain, 1996). Therefore, the activity of GSH-Px can be expressed by the reaction speed of GSH-Px catalyzing the reaction of hydrogen peroxide with reduced glutathione (GSH) to produce water and oxidized glutathione (Lawrence and Burk, 1978). GSH-Px activity (U/mL) is defined as the amount of enzyme per milliliter of serum required to decrease the GSH concentration by 1 μmol/L per minute.

The contents of immunoglobulin M (IgM), IgG, TNF-α, IL-6, IL-1β, interferon γ (IFN-γ), and toll-like receptor 4 (TLR4) in serum samples were determined using enzyme-linked immunosorbent assay kits (Nanjing Jiancheng Bioengineering Institute, Nanjing, Jiangsu, China) and expressed as the gram per mL of serum. The operating procedure was carried out according to the manufacturers’ instructions.

#### Determination of the Mucosal and Liver Parameters

Approximately 0.3 g mucosal or liver samples were homogenized (1:9, wt/vol) with ice-cold 0.86% sodium chloride solution using an Ultra-Turrax homogenizer (Tekmar Co., Cincinnati, OH, United States) and centrifuged at 3,000 rpm for 15 min at 4°C. The resultant supernatant was collected and immediately stored at −20°C for subsequent analysis. Secretory IgA (sIgA) content in mucosal samples was determined by enzyme-linked immunosorbent assay using microtiter plates and chicken-specific sIgA quantitation kits (Nanjing Jiancheng Bioengineering Institute, Nanjing, Jiangsu, China). Results are expressed as the μg per mg of protein. The total protein concentration of mucosa and liver was also determined according to the method described by Bradford using bovine serum albumin as the standard protein (Bradford, 1976). The content of IgG, IgM, TNF-α, IL-6, IL-1β, IFN-γ, and TLR4 in the liver and mucosa were measured according to the method described above and expressed as the μg per mg of protein.

### Statistical Analysis

Data were analyzed by one-way analysis of variance (ANOVA) using SPSS (2008) statistical software (Ver. 16.0 for windows, SPSS Inc., Chicago, IL, United States). Differences in means among treatment groups were separated using the least significant difference (LSD). *P* values less than 0.05 were considered indicative of statistical significance with 95% confidence.

## Results

### Antioxidant Activity Evaluated by H_2_O_2_-Induced Injury Cell Model

#### Effects of IDPG on the Viability of RAW264.7 Cells Treated With or Without H_2_O_2_

**FIGURE 1:**
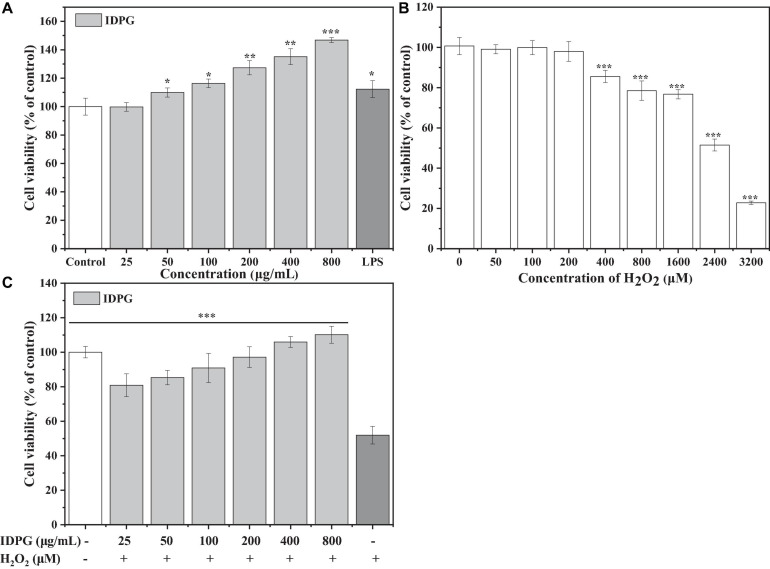
**(A)** Effects of incomplete degradation products of galactomannan (IDPG) on the cell viability of RAW264.7 cells. The RAW264.7 cells were treated with IDPG for 24 h; All data were in triplicate, and significance was determined by ANOVA; *P < 0.05, **P < 0.01, ***P < 0.001 as compared to the untreated control. **(B)** Effect of H_2_O_2_ on cell viability. Cells were treated with H_2_O_2_ for 4 h; All data were in triplicate, and significance was determined by ANOVA; ***P < 0.001 as compared to the untreated control. **(C)** Protective effect of IDPG on the H_2_O_2_ injured RAW 264.7 cells. Cells were treated with IDPG for 24 h before incubation with H_2_O_2_ (2.4 mM) for 4 h. All data were in triplicate, and significance was determined by ANOVA; ***P < 0.001 as compared to the H_2_O_2_ model group.

As shown in Figure 1A, the IDPG significantly promoted the proliferation of RAW264.7 cells in a concentration-dependent manner (*P* < 0.05). When the concentration of IDPG was 200–800 μg/mL, the relative survival rate of RAW264.7 cells even exceeded the positive control LPS. Regarding H_2_O_2_, it can cause RAW264.7 cells to lose viability, which was more pronounced at high concentrations (Figure 1B). After culturing with 2.4 mM H_2_O_2_ for 4 h, the survival rate of the cells was 51.51 ± 2.97% of the control group. Therefore, 2.4 mM H_2_O_2_ was selected to culture the cell for 4 h to investigate the protective effects of IDPG in the system. Compared with the H_2_O_2_ model group, the cells cultured with IDPG showed a higher survival rate (*P* < 0.05), and it was concentration-dependent (Figure 1C).

#### Effect of IDPG on the Level of MDA and SOD in Cells

**FIGURE 2:**
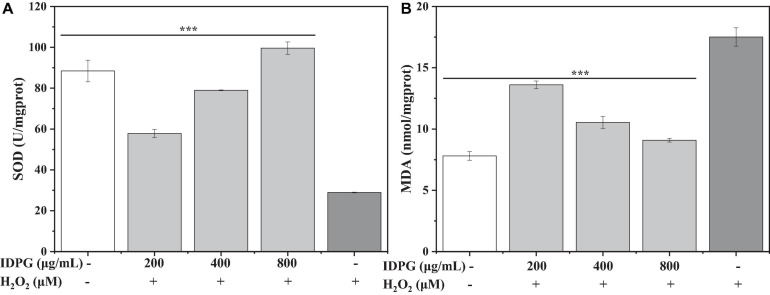
Effects of incomplete degradation products of galactomannan (IDPG) on the production of superoxide dismutase (SOD) **(A)** and malondialdehyde (MDA) **(B)** in H_2_O_2_ injured RAW 264.7 cells. Cells were treated with IDPG for 24 h before the incubation with H_2_O_2_ (2.4 mM) for 4 h. Data were all in triplicate, and significance was determined by ANOVA; ***P < 0.001 as compared to the H_2_O_2_ model group.

As shown in Figure 2A, compared with the H_2_O_2_ model group, the SOD activity in cells cultured with IDPG increased significantly, and the difference was statistically significant (*P* < 0.05). Also, the SOD activity increased with the increase of IDPG concentration. As for MDA, its content is higher in the H_2_O_2_ model group than (17.51 ± 0.75 U/mg protein) in the control group (7.80 ± 0.35 nmol/mg protein). More importantly, the MDA content of the IDPG group was significantly lower than that of the H_2_O_2_ model group (*P* < 0.05), which decreased with the increase of the concentration, as shown in Figure 2B.

### Effect of IDPG on the Secretion of NO, TNF-α, IL-6, and TLR4 in Cells

**FIGURE 3:**
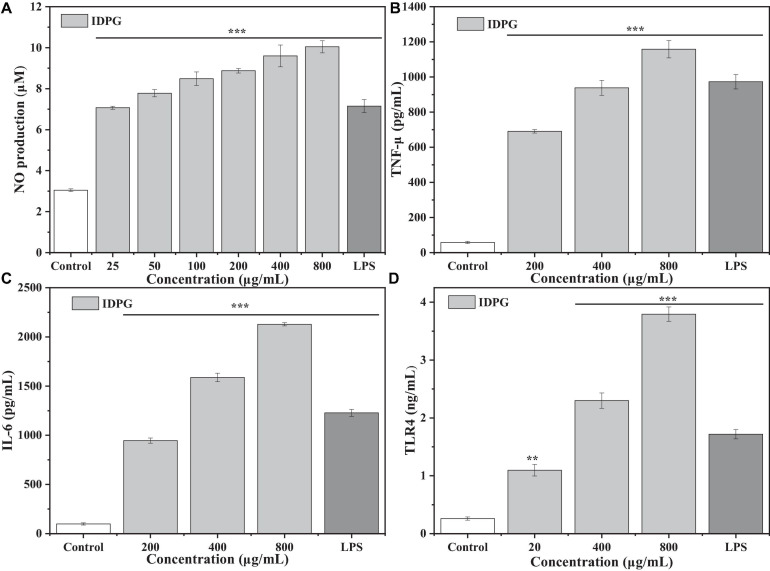
Effect of incomplete degradation products of galactomannan (IDPG) on the NO production **(A)** and the secretion of tumor necrosis factor-α (TNF-α) **(B)**, interleukin 6 (IL-6) **(C)**, and Toll-like receptor 4 (TLR4) **(D)** in RAW 264.7 cells. Cells were treated with IDPG for 24 h. Data were all in triplicate, and significance was determined by ANOVA; **P < 0.01, ***P < 0.001 as compared to the control group.

As shown in Figure 3, compared with the control group, the IDPG significantly increased the secretion of NO, TLR4, TNF-α, and IL-6 (*P* < 0.05) with a concentration-dependent relationship. The promotion effect on NO, TLR4, TNF-α, and IL-6 secretion was more pronounced when the IDPG concentration was 800 ug/mL and exceeded that of the LPS group.

### Effect of IDPG on the Antioxidant Function of Laying Hens

**FIGURE 4:**
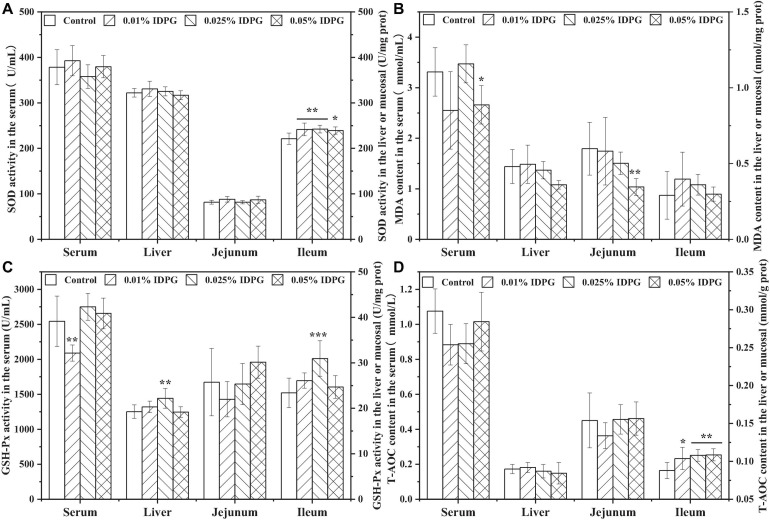
Antioxidants and malondialdehyde in different organs of laying hens fed diets supplemented with incomplete degradation products of galactomannan (IDPG). *P < 0.05, **P < 0.01, and ***P < 0.001 compared with control group in each tissue. **(A)** Superoxide dismutase (SOD); **(B)** Malondialdehyde (MDA); **(C)** Glutathione peroxidase (GSH-Px); **(D)** Total antioxidant capacity (T-AOC).

Analysis of the parameters concerning antioxidant capacity in the serum, liver, jejunum, and ileum are shown in Figure 4. The addition of IDPG into the diet of laying hens significantly increased SOD activity in the ileum (*P* < 0.05), yet no obvious change occurred on that in the serum, liver, and jejunum. MDA content in the serum and jejunum of the group supplemented with 0.05% IDPG was significantly lower than what was recorded for the control group (*P* < 0.05). However, there was no significant difference in the MDA content of the liver and ileum between the IDPG-supplemented group and the control group. Concerning the GSH-Px activity and contrary to the previous results of MDA content, GSH-Px activity was significantly increased (*P* < 0.05) in the liver and ileum of the laying hens fed diets supplemented with 0.025% IDPG. However, GSH-Px did not differ among the serum and jejunum with IDPG treatment. Additionally, among the tissues or organs (serum, liver, jejunum, and ileum) selected for testing, only the T-AOC content of ileum increased significantly (*P* < 0.05). Specifically, the T-AOC content in the ileum increased linearly with the increasing concentration of dietary IDPG supplementation (0.01, 0.025, and 0.05%) relative to the control group.

### Effect of IDPG on the Immune Function of Laying Hens

**FIGURE 5:**
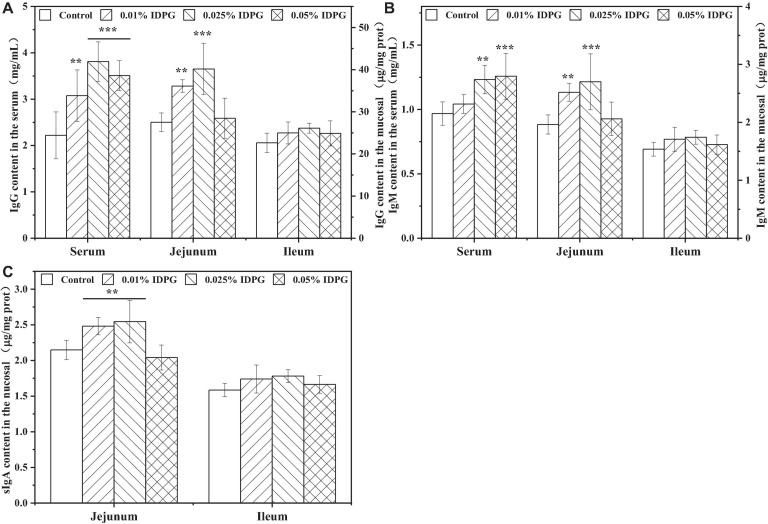
Immunoglobulins in the serum, jejunum and ileum of laying hens fed diets supplemented with incomplete degradation products of galactomannan (IDPG). **P < 0.01 and ***P < 0.001 compared with control group in each tissue. **(A)** Immunoglobulin G (IgG); **(B)** IgM; **(C)** secretory IgA (sIgA).

Figure 5 displays the contents of immunoglobulins in the serum, jejunum, and ileum of laying hens receiving diets supplemented with and without IDPG. As seen, contents of IgG and IgM in the serum and jejunum were significantly increased by the introduction of IDPG additives in the diet of laying hens (*P* < 0.05). Also, IDPG supplementation increased (*P* < 0.05) the content of secretory IgA (sIgA) in the jejunum. Additionally, in the ileum, laying hens fed diets containing IDPG showed higher content of IgG, IgM, and sIgA than that fed without IDPG. However, the noted difference was not statistically significant.

**FIGURE 6:**
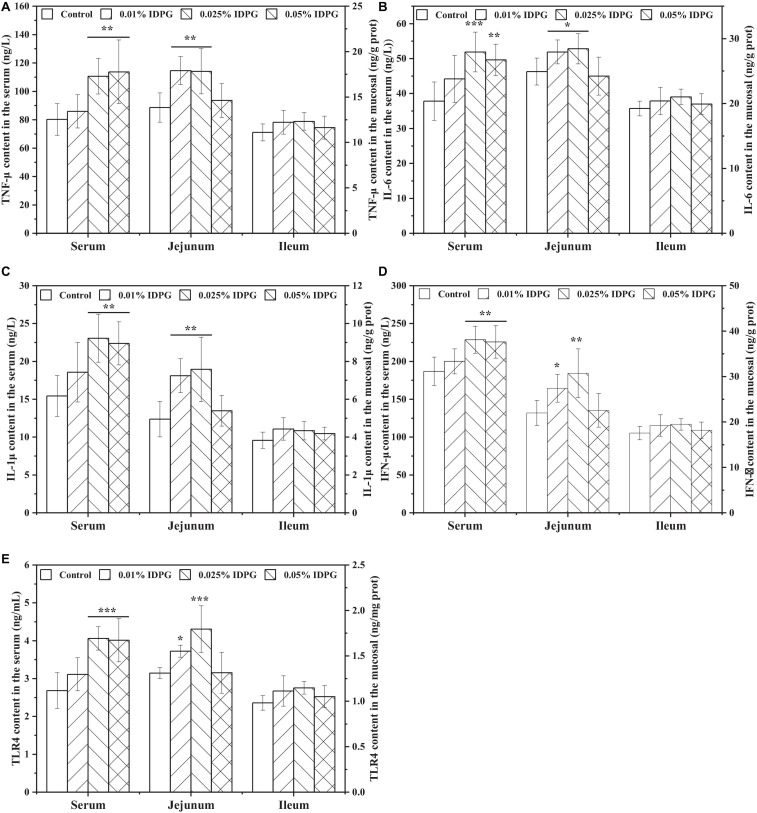
Effects of incomplete degradation products of galactomannan (IDPG) on the secretion of tumor necrosis factor-α (TNF-α) **(A)**, interleukin 6 (IL-6) **(B)**, IL-1β **(C)**, interferon γ (IFN-γ) **(D)**, and toll-like receptor 4 (TLR4) **(E)** in the serum, jejunum and ileum of laying hens. *P < 0.05, **P < 0.01, and ***P < 0.001 compared with control group in each tissue.

Quantification of TNF-α, IL-6, IL-1β, IFN-γ, and TLR4 in the serum, jejunum, and ileum are displayed in Figure 6. Similar to the results of immunoglobulins, dietary IDPG supplementation (0.025% and 0.05%) significantly increased the contents of TNF-α, IL-6, IL-1β, IFN-γ, and TLR4 (*P* < 0.05) in the serum. As for the jejunum, contents of the quantified cytokines increased with the addition of IDPG (0.01% and 0.025%) into the basal diets. However, contents of TNF-α, IL-6, IL-1β, IFN-γ, and TLR-4 in the ileum did not vary statistically among treatments.

## Discussion

Natural polysaccharides and their degradation products have been investigated as antioxidant enhancers and immunomodulators in recent decades. Herein, IDPG was chosen to investigate to understand its positive effects on the antioxidant and immune system of RAW264.7 cells and aged laying hens. Generally, the possible routes by which natural polysaccharides and their degradation products can improve the antioxidant capacity of the body are as follows: (1) activate the antioxidative enzymes such as glutathione peroxidase (GSH-Px) and superoxide dismutase (SOD) through metabolic pathways; and (2) block oxidative chain reactions attributed to lipid peroxide, and eventually inhibit the peroxidation of unsaturated fatty acids on its membrane structure. In the investigation of the antioxidant system, the free radical theory is an important theory involving free radicals (Hong and Liu, 2004; Dong et al., 2020; Wang et al., 2020). They are a kind of intermediate product formed in the metabolic process of animals. Excessive free radicals in the animal’s body will destroy the barrier function of the intestines, cause protein denaturation, block cell division, and affect metabolism and cause diseases. Fortunately, antioxidant active substances can protect the body from the damage of free radicals. Antioxidants in the body can reduce the incidence of diseases. Examples of important antioxidants include SOD and GSH-Px (Dong et al., 2020). The antioxidant enzymes SOD and GSH-Px are considered to be the main elements of the first level of antioxidant defense in a cell because they form a major protective system against oxidative damage. Through *in vitro* experiment, the model of H_2_O_2_-injured cells was introduced, which is a common model to evaluate the antioxidant capacity of the sample tested (Holmström and Finkel, 2014). In a variety of cellular assays, the IDPG was demonstrated to attenuate H_2_O_2_-induced oxidative stress injury in macrophages, as shown by the promoted SOD activity and the decreased malondialdehyde (MDA) levels. Wherein, the MDA is a major lipid peroxidation product derived from oxygen radicals attacking polyunsaturated fatty acids in biofilms. The MDA content can reflect the degree of lipid peroxidation and indirectly reflect the extent of cell damage. Consistent with our findings, Germano et al. (2019) found that compared with the uncoated control, the galactomannan-carnauba wax coating increased the SOD activity of guava. Based on these results, the experiments *in vivo* were further conducted in laying hens to study the effect of IDPG on the antioxidant function.

In the evaluation of the antioxidant capacity of IDPG on laying hens, total antioxidant capacity (T-AOC) and GSH-Px activity were also evaluated except SOD. T-AOC is a helpful representation of the overall level of enzymatic as well as non-enzymatic antioxidants (Surai, 1999). Our results showed that supplementation with IDPG to the basal diet of laying hens increased levels of SOD, GSH-Px, and T-AOC. In agreement with our findings, Bozkurt et al. (2016) found that laying hens fed with yeast cell wall manno-oligosaccharide (MOS) showed a significant improvement in SOD activity in the liver, but not GSH-Px content. However, in the present study, the effect of increasing antioxidants levels differs in the four tissues or organs (serum, liver, jejunum, and ileum), which is more pronounced in the ileum.

In addition to antioxidants, we also tested MDA contents. In this work, 0.05% IDPG dietary supplementation significantly lowered MDA content in the serum and jejunum relative to the control group, while no significant difference was observed in the MDA content of the liver and ileum. Consistent with our findings, Bozkurt et al. (2016) revealed that the usage of yeast cell wall MOS to the diet of laying hens has no significant effect on MDA content in the liver. Another report involving acute alcohol-intoxicated mice found that partially hydrolyzed guar gum had a significant effect on the reduction of MDA content in the serum (Wu et al., 2019). Unfortunately, in the same tissue or organ, the scavenger roles of these antioxidative enzymes for preventing oxidative damage did not play a significant role in reducing MDA content. For example, the promotion of the level of SOD, GSH-Px, and T-AOC in the ileum response to IDPG supplementation did not result in a reduction in MDA content. A possible reason for this discrepancy could be that IDPG supplementation mainly reduces the formation of free radicals by increasing the content of antioxidants (Captions and Document, 1997). Therefore, more antioxidants mean an overall decrease in free radicals which explains why we did not see the MDA content drop. To conclude, IDPG mainly improved the antioxidant capacity by increasing the antioxidants contents (GSH-Px, SOD, and T-AOC) and secondly by reducing the MDA content. The elevation of the contents of these above-mentioned antioxidants may help in boosting the immune system, based on the cognition that the immune cell functions are strongly influenced by the antioxidant/oxidant balance. Moreover, the immune system cells usually have a high percentage of polyunsaturated fatty acids in their plasma membrane, which are very much susceptible to oxidative attack resulting in highly damaging lipid peroxidation. Therefore, it may be necessary that these cells contain higher concentrations of antioxidants than do other cells (Knight, 2000).

After revealing the ability on enhancing antioxidant function, the ability of IDPG on regulating immune function was next evaluated. The immunoglobulins and cytokines contents were emphatically assessed because immunoglobulins and cytokines play a critical role in humoral immunity and cell-mediated immunity, respectively, which can closely reflect the effect of any tested materials on the immune system (Ferreira et al., 2015). As an important performer toward humoral immunity, immunoglobulins such as IgA, IgM, and IgG can specifically bind to antigens to resist the invasion of pathogenic bacteria (Su et al., 2013). As shown in Figure 5, the inclusion of IDPG into the basal diet of laying hens significantly increased the content of IgG and IgM in the serum and jejunum. Consistent with the present findings, Yin et al. (2008) reported that the usage of 0.2% galactomanno-oligosaccharide can increase IgG, IgM, and IgA content in the serum of early-weaned piglets. Similarly, Zhou et al. (2019) reported that the addition of 0.1% MOS, obtained from degraded *Konjac* glucomannan, increased jejunum IgG and IgM contents in broilers after 21 days. Moreover, the sIgA content in the jejunum of laying hens supplemented with IDPG (0.01 or 0.025%) was higher than that of the control birds. As the most secreted immunoglobulin in the body, sIgA is the first effective defense against pathogens that invades mucosa. sIgA can effectively combine with pathogenic microorganisms to form an immune complex and prevent the adhesion of pathogenic microorganisms to mucosal epithelial cells (Fujioka et al., 1998; Robinson et al., 2001). Furthermore, the resultant complex can stimulate goblet cells in intestinal mucosa to secrete a large amount of mucus, thus reducing the contact between pathogenic microorganisms and intestinal mucosa (Rodríguez et al., 2006). Based on the fact that it is almost impossible for serum immunoglobulin to reach the intestine and function, the observed significant increase of sIgA content in the jejunum is important because the increased antibodies can cover the surface of the intestinal mucosa to protect villi from damage (Fujioka et al., 1998). Gómez-Verduzco et al. (2009) observed a similar result in which a significant increase in the mucosal concentration of IgA was observed with 0.05% yeast cell wall MOS supplementation to a broiler diet. It seems that supplemental IDPG could effectively suppress enteric pathogens by promoting the immune system and its responses, as well as improving the integrity of intestinal mucosa. A similar mechanism of benefit was proposed by Spring et al. (2000).

Cytokine, a small molecule polypeptide, is synthesized or secreted by a variety of tissue cells (mainly immune cells). Examples of cytokines include interleukins, interferons, tumor necrosis factors, and more. Cytokines can mediate intercellular interactions while also serving a variety of biological functions, such as cell growth promotion, immune response regulation, participation in inflammatory responses, and inhibition of tumor growth (Bai et al., 2012). In this experiment, we selected TNF-α, IL-6, IL-1β, IFN-γ, and TLR4 for measurement. The reason why we chose these is that they are reported to be related to galactomannan (Hernandez et al., 2011). Results showed that supplemental IDPG remarkably improved secretion of TNF-α, IL-6, IL-1β, and IFN-γ in the serum and jejunum. Consistent with our findings, Yin et al. (2008) found that supplementation of 0.2% galactomanno-oligosaccharide into the diet of early-weaned piglets up-regulates serum IL-1β and IL-6 level. Furthermore, *in vitro* experiments of IDPG also showed its capacity for promoting effective activation of RAW264.7 cells and enhancing the secretion of NO, TNF-α, IL-6, and TLR4 (*P* < 0.05).

Amongst the several cytokines tested, a significant increase in IL-1β response to IDPG supplementation was notable due to IL-1β being necessary for inducing a specific adaptive anti-tumor response (Ghiringhelli et al., 2009). More importantly, IDPG-induced secretion of IL-6 and TNF-α may help establish an inflammatory environment that can initiate or maintain the activation of DC that can then activate naive T cells. Meantime, TNF-α may be involved in inducing tumor cells to die (Aymeric et al., 2010; Van Herreweghe et al., 2010). Moreover, the secretion of IFN-γ significantly increased with the addition of IDPG. This finding may be because IFN-γ is the main executive cytokine of T helper lymphocytes (Th1 cells), and mannan derivatives are conducive to T cell differentiation into Th1 cells (Sheng et al., 2006). The measurement of TLR4 revealed that the usage of IDPG significantly increased the content of TLR4 in the serum and jejunum. It has been reported that TLR4 has a high affinity for acemannan (Karaca et al., 1995), and galactomannan and acemannan share a backbone composed of mannose (differing only in side-chain configuration). Therefore, the noted high-secretion of cytokines in line with the increase in TLR4 content indicates that IDPG may activate TLR4 signaling pathways, triggering the maturation of DCs or other immune cells, all of which elicits an immune response that leads to the stimulated secretion of IgG, IgM, sIgA, TNF-α, IL-6, IL-1β, and IFN-γ.

## Conclusion

In the present study, the IDPG was prepared from the *Sesbania cannabina* seeds, and its antioxidant and immunomodulatory activity was evaluated *in vitro* and *in vivo*. Through measuring SOD and MDA, the IDPG showed a stronger protective effect against H_2_O_2_-induced oxidative damage *in vitro* using macrophage RAW264.7 cell as a model. Moreover, IDPG treatments in basal feeds for laying hens showed higher levels of SOD, GSH-Px, and T-AOC relative to the control group. However, there exist disparities between the extent of beneficial improvements between the four tissues or organs analyzed (serum, liver, jejunum, and ileum). Usage of 0.05% IDPG significantly lowered the MDA content in the serum and jejunum but had no significant effect on the MDA content in the liver and ileum. More importantly, IDPG supplementation to the basal diet of laying hens could promote the secretion of IgG, IgM, sIgA, TNF-α, IL-6, TLR4, IL-1β, and IFN-γ. This result was consistent with the effect of IDPG on RAW264.7 cells by the evaluation of NO, TNF-α, IL-6, and TLR4. These results indicated that IDPG can be used as an ideal additive to enhance antioxidant and immune function, thereby boosting the health of laying hen. More importantly, the development of *S. cannabina* seeds into feed additives with outstanding antioxidant and immune activity will promote its economic value.

## Data Availability Statement

The original contributions presented in the study are included in the article/supplementary material, further inquiries can be directed to the corresponding author/s.

## Ethics Statement

The experimental protocols used in this experiment, including animal care and use, were reviewed and approved by the Animal Care and Use Ethics Committee of Nanjing Agricultural University (Nanjing, China). Written informed consent was obtained from the owners for the participation of their animals in this study.

## Author Contributions

YT: investigation. TW and CL: supervision. ZL and YZ: writing – original draft. QY: writing – review and editing. All authors contributed to the article and approved the submitted version.

## Conflict of Interest

The authors declare that the research was conducted in the absence of any commercial or financial relationships that could be construed as a potential conflict of interest.
